# Sero-Prevalence Surveillance to Predict Vaccine-Preventable Disease Outbreaks; A Lesson from the 2014 Measles Epidemic in Northern Vietnam

**DOI:** 10.1093/ofid/ofz030

**Published:** 2019-01-24

**Authors:** Marc Choisy, Son Tung Trinh, Thi Ngoc Diep Nguyen, Tran Hien Nguyen, Quynh Le Mai, Quang Thai Pham, Nhu Duong Tran, Duc Anh Dang, Peter W Horby, Maciej F Boni, Juliet Bryant, Sonia O Lewycka, Behzad Nadjm, H Rogier Van Doorn, Heiman F L Wertheim

**Affiliations:** 1 Oxford University Clinical Research Unit, Wellcome Trust Asia Programme, Hanoi, Vietnam; 2 MIVEGEC (UMR CNRS, IRD & University of Montpellier), Montpellier, France; 3 National Institute of Hygiene and Epidemiology, Hanoi, Vietnam; 4 Nuffield Department of Clinical Medicine, University of Oxford, Oxford, UK; 5 Oxford University Clinical Research Unit, Wellcome Trust Major Overseas Programme, Ho Chi Minh City, Vietnam; 6 Center for Infectious Disease Dynamics, Department of Biology, Pennsylvania State University, State College, Pennsylvania; 7 Department of Medical Microbiology and Radboudumc Center for Infectious Diseases, Radboudumc, Nijmegen, the Netherlands

**Keywords:** measles, seroprevalence, vaccination, Vietnam

## Abstract

**Background:**

During the first half of 2014, a severe outbreak of measles occurred in northern Vietnam, causing 15 033 confirmed cases and 146 deaths.

**Methods:**

To evaluate the population-level seroprevalence of protection against measles in the period before the outbreak, we made use of an existing age-stratified serum bank, collected over the year before the outbreak, between November 2012 and December 2013, from 4 sites across the country (Hanoi, Hue, Dak Lak, and Ho Chi Minh City). Data from the UNICEF’s Multiple Indicator Clustered Surveys (MICS), carried out in Vietnam during the first quarter of 2014, were used to assess the vaccine coverage in 6 ecological regions of Vietnam.

**Results:**

Results revealed a large discrepancy between levels of protection, as estimated from the serology and vaccine coverage estimated by UNICEF’s MICS. Variation in seroprevalence across locations and age groups corresponded with reported numbers of measles cases, most of which were among the 0–2-year-old age group and in the northern part of the country.

**Conclusions:**

Our study presents a strong case in favor of a serosurveillance sentinel network that could be used to proactively tune vaccination policies and other public health interventions.

Measles is a highly transmissible infection that causes high morbidity and mortality, particularly in malnourished or otherwise compromised children [1–3]. In the prevaccination era, more than 90% of individuals contracted the disease by the age of 10 [4]. Since the initiation of the Expanded Programme on Immunization (EPI) in 1974 by World Health Organization (WHO), the worldwide incidence of measles has substantially declined [5]. In 2000, pursuing the goal of global measles eradication, the WHO prioritized vaccination campaigns in the 45 countries that together accounted for more than 90% of estimated global measles deaths [6]. The WHO strategy for measles reduction is based on achieving vaccine coverage of >90% for 2 doses of vaccine, delivered at 9 and 18 months [3, 7]. About 15% of children injected with a single dose of a measles virus–containing vaccine (MCV) at the age of 9 months fail to develop immunity [6, 8, 9]. A second dose at the age of 18 months is thus necessary and confers approximately 98% protection [9, 10].

Despite the efforts deployed toward global eradication, measles remains a leading cause of vaccine-preventable childhood mortality in developing countries, with the greatest incidence occurring in children younger than 2 years of age [11–13]. Measles has recently re-emerged in a number of countries in Europe and North America due to refusal to vaccinate [14]. Insufficient vaccine uptake has been identified as the main cause of re-emergence [15]. The resilient circulation of misinformation regarding associations between vaccination and autism and other adverse events, against a backdrop of perceived decreased hazard of measles due to successful reductions in transmission [16], is a major impediment to maintaining high vaccination rates [17, 18]. As measles is highly infectious—with R_0_ estimates between 12 and 18, depending on the population considered [19]—a small proportion of seronegative (unprotected) individuals could be sufficient to start and sustain an epidemic [11, 20].

Vietnam is 1 of the 45 countries that implements the WHO’s EPI. Despite high officially reported vaccination coverage rates, Vietnam experienced a severe measles outbreak in 2014 (more than 15 033 confirmed cases and 146 fatalities) [21], despite high official vaccine coverages (>90% for all provinces and >95% for most of them). During the 2014 outbreak, most children admitted to hospitals with measles infection were younger than 12 months, and many were therefore not fully vaccinated. However, even among children older than 12 months who presented to hospitals with measles, a large proportion did not have a history of vaccination. The epidemic appeared to be more severe in the north of the country, with 763 reported cases in Ho Chi Minh City (south, official population 8.4 million), vs 4216 in the capital city of Hanoi (north, official population 3.4 million).

Here, we aimed to estimate the vaccine coverage and serum protection levels by age and region just before the 2014 epidemic, in order to assess whether any of these estimates is more in accordance with the observed magnitude of the epidemic than the official vaccine coverage numbers. Population protection levels were estimated from an existing age-stratified serum bank that had been collected between November 2012 and December 2013 from 4 hospitals across Vietnam. Vaccination coverages were estimated in 6 regions of Vietnam from the immunization data from the UNICEF Multiple Indicators Clustered Survey (MICS), conducted in Vietnam in 2014 (General Statistical Office and UNICEF 2015).

## METHODS

### Serum Samples Collection

**Figure 1.**
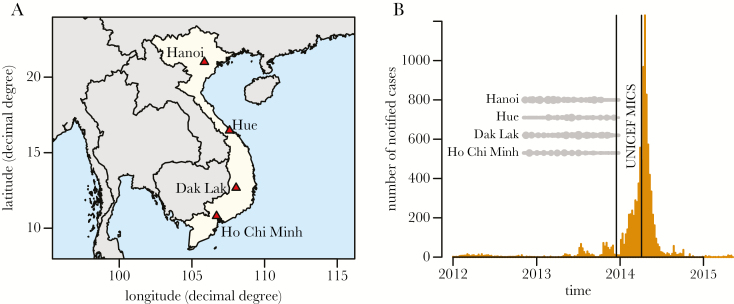
A, Map showing locations of the 4 sampled hospitals in Vietnam: Hanoi, Hue, Dak Lak, and Ho Chi Minh City. B, Weekly measles notifications for the 2014 epidemic in Vietnam. Gray dots show the timing of serum sampling in the 4 sampling sites (see Supplementary Figure 1 for more details), the two vertical lines show the timing of the UNICEF Multiple Indicator Clustered Surveys.

The National Institute for Hygiene and Epidemiology initiated a serum biobank project in 2012, comprising periodic age-stratified residual serum collections from 4 hospitals in Vietnam (Thanh Nhan Hospital in Hanoi, Hue Central Hospital in the city of Hue [central coastal region]), Dak Lak General Hospital in the central highlands city of Buon Ma Thuot, and Gia Dinh People’s Hospital in Ho Chi Minh City (Figure 1A). We used a total of 3662 serum samples from this biobank, representing children and women of childbearing age. Our sampling protocol was based on recommendations from the European Sero-Epidemiology Network (ESEN), which suggests testing at least 50 samples per gender and suggests a 1-year age band for individuals younger than 20 and a 5-year age band for individuals older than 20 [22]. Given that most measles cases in the 2014 Vietnam outbreak were infants and children, we focused our testing on the younger age classes and aimed at testing 50 samples per gender with a 1-year age band for individuals younger than 10 years old. In addition, we tested serum from women of childbearing age, using 50 samples per 1-year age band from age 16 to 20 years and 100 samples per 5-year age band from age 20 to 35 years.

### Incidence

Line listing incidence data of confirmed cases were retrieved from the WHO EPI in Vietnam and aggregated by province, gender, age, and week.

### Serology

Measles IgG ELISA assays (SERION Immunologics, Würzburg, Germany) were conducted on all subsampled sera according to the manufacturer’s instructions [23]. The test used 10 µL of serum, and titers were expressed in IU/L based on quantitative spectrophotometry at 620–690 nm. Titers <200 IU/L indicated susceptibility; titers >275 IU/L indicated protection; intermediate titers were designated equivocal. To counter the difficulties in the analysis of equivocal results, analyses were conducted using both thresholds. Reported sensitivity and specificity of the assay were 99% and 93%, respectively, according to the manufacturer.

### Population Protection

Percentages of immunized people per location, gender, and age class were derived from IgG levels, considering both the 200- and 275-IU/L thresholds. Effects of age class and location (and gender when possible), as well as interactions between age and location (and age and gender when possible), on the percentages of immunized individuals were modeled by multivariate logistic regressions with a logit link [24]. Age bands were coded as factors (instead of numeric variables) in order to favor flexibility in the relationship between age and seroprevalence (at the risk of lower statistical power). Statistical significance was assessed by likelihood ratio tests (LRTs) in order to control for potential confounding effects [25] that may persist despite our efforts for a balanced sampling scheme (Supplementary Figure 2 and Supplementary Table 1. For both the 200- and 275-IU/L thresholds used to calculate seroprevalence, the model was run twice: once on all the data and once on the under-10 age classes. In the first case, gender was not introduced as a covariate, whereas gender and age × gender interactions were introduced as covariates in the second case. All the analyses were performed in R, version 3.3.2 [26].

### Vaccination Coverage

The UNICEF MICS is a stratified clustered survey. The strata are the urban and rural areas within the 6 ecological regions of Vietnam (12 strata in total). Within each stratum, a number of census enumeration areas (considered clusters) was randomly selected with probability proportional to size (510 selected enumeration areas in total), and within each enumeration area, 20 households were drawn at random. Household interviews using structured questionnaires were completed between December 16, 2013, and April 5, 2014. Questions were asked on different topics, including immunization. After the UNICEF protocol, the vaccine coverage was computed from the answers to questions M3MD, IM16, and HF13MD regarding, respectively, (1) the day of measles vaccination from the immunization card, (2) measles vaccination information reported by the mother, and (3) the day of measles vaccination from the health facility. Immunization data were only available for children below the age of 3 years. See the Supplementary Data for more details on the questions (Supplementary Tables 2–4) and the algorithm. Analyses were performed with the survey R package, version 3.33-2 [27].

## RESULTS

**Figure 2.**
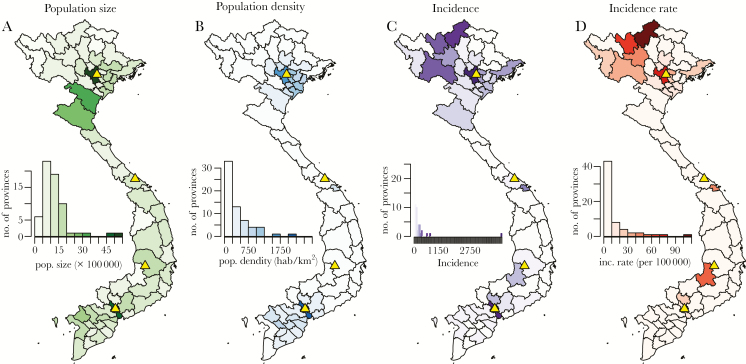
A, B, Population size (A) and density (B) for Vietnam as from the 2009 population and housing census [34]. C, D, Incidence (C) and incidence rate (incidence divided by population size) (D) for the 2014 epidemic, as reported by the World Health Organization’s Expanded Programme on Immunization. Yellow triangles show the 4 serum sampling sites of 2013 (Figure 1A).

The numbers of collected samples per location, gender, and age class are shown in Supplementary Table 1 and Supplementary Figure 2. Supplementary Figure 1 further shows the times of sample collections in the 4 locations. Given that the 2014 epidemic occurred during the first 6 months of 2014, our samples give a snapshot of the situation during the 12 months that preceded the epidemic (Figure 1B). Most of the cases recorded by the WHO EPI occurred among infants and young children (38% of cases below the age of 1 year and 11% of cases age 1–2 years) (Supplementary Figure 3) and in northern Vietnam (4216 cases in the province of Hanoi only) (Figure 2).

Table 1 summarizes the results of the likelihood ratio tests on the effects of covariates and interactions in the logistic regressions, considering the 2 density thresholds (200 and 275 IU/L) and the 2 age ranges (all ages and under 10 years old). These models show strong age and location effects and an absence of gender effects. Furthermore, there are significant age × location interactions, except when considering the 275-IU/L threshold for children under 10.

**Table 1.** Likelihood Ratio Tests of the Effects of Covariates and Interactions (“x”) in the Logistic Regressions Explaining Seroprevalence, Considering the 200 and 275 IU/L Thresholds and Stratified by Age <10 Years or for All Ages

			200 IU/L		275 IU/L	
		df	Deviance	*P* (>chi)	Deviance	*P* (>chi)
Age <10 y	Location	3	25.989	<2 × 10^-16^	20.172	.0002
	Gender	1	0.074	.7861	1.272	.2593
	Age	9	54.181	<2 × 10^-16^	44.348	<2 × 10^-16^
	Location × age	27	44.060	.0204	35.697	.1221
	Gender × age	9	9.761	.3702	10.017	.3491
All ages	Location	3	25.599	<2 × 10^-16^	27.340	<2 × 10^-16^
	Age	16	283.472	<2 × 10^-16^	286.606	<2 × 10^-16^
	Location × age	48	91.853	.0001	79.733	.0027

**Figure 3.**
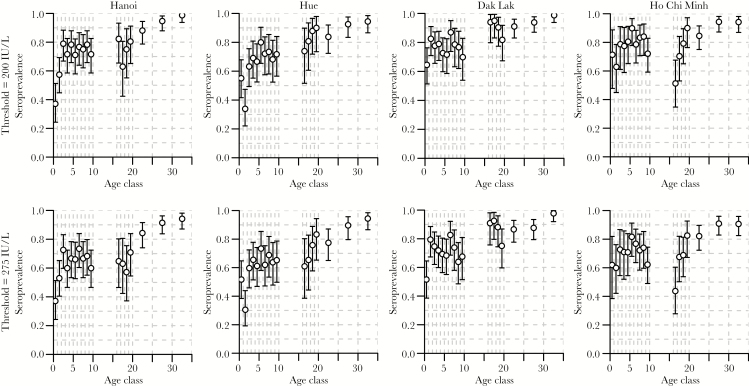
Estimated levels of seroprevalence (circles) together with 95% confidence intervals (error bars) by age class in years (x-axes) for the 200- and 275-IU/L thresholds (first and second rows, respectively) for the 4 locations (by columns). Vertical dashed gray lines show the age classes.

Figure 3 illustrates the relationships between estimated levels of seroprevalences and age for the 4 locations (by column) and the 2 thresholds (by row). The shapes of the relationships were qualitatively similar for the 2 thresholds, as well as for the 4 locations: increase in seroprevalence with age for the age classes 0–1, 1–2, and 2–3, stabilization of seroprevalence levels until the age of 10 years, slight decrease during the teenage years and increase again in the early 20s, after which seroprevalences reach levels above 90% (after 25 years old) and 95% (after 30 years old). There were, however, some large quantitative differences between the 4 locations: Hanoi and Hue show particularly low levels of seroprevalence for the age bands 0–1 and 1–2 (particularly low for the 1–2-year-old age class in Hue). Such extremely low levels of prevalence (approximately 50%) for these age bands were not observed in Dak Lak or Ho Chi Minh City, where the levels were closer to 70%. Seroprevalence for the 16–17 age band was particularly low in Ho Chi Minh City, and seroprevalence for the 16–19 age bands was particularly high in Dak Lak.

**Figure 4.**
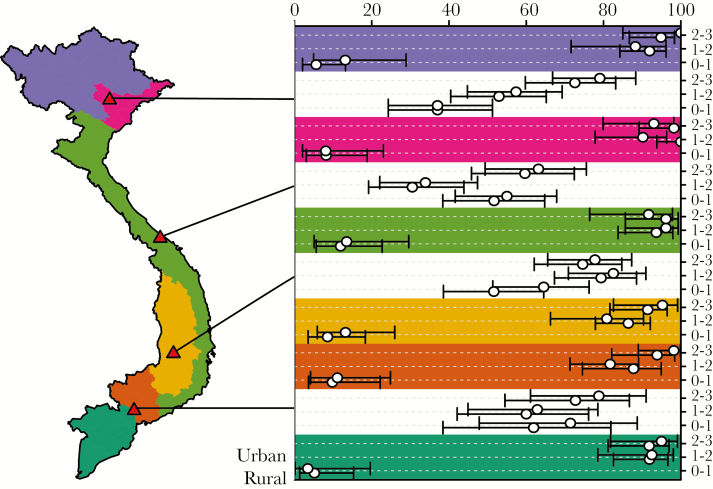
Population protection and vaccine coverage against measles in 2013–2014. The map on the left shows the 6 ecological regions of Vietnam, color-coded, together with the serum sampling sites (red triangles) (Figure 1A). The chart on the right shows the estimates (circles) with 95% confidence intervals (horizontal bars) of the seroprevalence (white background), taken from Figure 3, and the vaccine coverage (colored background), increasing from 0% to 100% from left to right, and ordered by age class from bottom to top (see the legend on the right). For the seroprevalence estimates, both the 275-IU/L (bottom) and 200-IU/L (top) thresholds are shown for each age class. For the vaccine coverage estimates, both the rural (bottom) and urban (top) strata are shown for each age class.

The estimates of population protection for the age classes <3 are extracted from Figure 3 and shown in Figure 4 (with white background, with thresholds 275 IU/L [bottom] and 200 IU/L [top]), together with the estimates of vaccine coverages from the UNICEF MICS (with a colored background mapping the 6 different regions of Vietnam), also for the age classes <3. Estimates of vaccine coverages are made separately for the rural (bottom) and urban (top) areas. This figure shows that seroprotection in the age class <1 (between 40% and 60%) is much higher than what can be expected from vaccination (25%, given a first shot at 9 months), even accounting for a vaccine efficacy of 85% for the first shot, probably due to maternal immunity in the first few months of life. On the contrary, vaccine coverage for this age class (around 10%) is much lower than the expected 25%. Looking at the 2 other age classes, it appears that vaccine coverages are quite high (around 95%), which is inconsistent with the corresponding observed levels of seroprevalence (between 50% and 80%).

## DISCUSSION

The data revealed an overall very low level of protection in the 4 study sites, with seroprevalence between 40% and 80% for children younger than age 5 years and levels of seroprevalence reaching 90% and 95% for ages 25 and 30 years, respectively. Such levels of protection were not adequate to provide efficient protection against epidemic measles transmission; previous modeling efforts have consistently recommended a minimum of 95% immunization to achieve herd immunity [19]. For the 0–1-year age class, the vaccination coverages estimated from the UNICEF MICS data are in line with the levels of population protection estimated from our serum bank, taking into account that the first vaccine dose is injected at the age of 9 months and that maternal immunity confers protection until the age of 6 months [28]. For the other age classes, however, our results highlight a marked discrepancy between the estimated levels of population protection (60%–70%) and vaccine coverages (90%–95%). The exact cause of this discrepancy is unknown and would require further in-depth investigation that is beyond the scope of this study.

Identifying the precise causes of the discrepancy between officially reported vaccine coverage rates and our estimated levels of protection is a priority to improve current immunization programs in Vietnam. Further specific investigations are needed to examine both the logistics and quality control of vaccine distribution, especially in regards to cold chain [29] and immune responses of vaccination [28]. However, whatever the causes of the discrepancy between reported vaccine coverage rates and our estimates of population immune protection, our results indicate that estimates of vaccine coverages like UNICEF MICS are poor predictors of the actual population protection. Measles being extremely contagious (R_0_ between 12 and 18), complete eradication remains a very difficult target in many countries. A number of strategies have been explored recently to improve the efficacy of vaccination campaigns. Outbreak response vaccination (ORV), for example, is a case-triggered policy that has been proposed based on the fact that measles vaccination can confer immunity shortly after injection and even mitigate the disease severity and transmission in subjects already infected [30–32]. A recent theoretical study proposes to go a step further in planning vaccination campaigns triggered not only by incidence but also by population seroprevalence, thus aiming to prevent outbreaks before they start [33]. The results of this study show that small-scale serological surveillance can greatly improve the efficacy of vaccination policies in low-incidence contexts (in high-incidence contexts, classical case-triggered strategies remain more efficient). In the context of Vietnam, where incidence levels overall remain low, a serology-based surveillance system could be the ideal complement to the current vaccination strategy, especially in light of our results showing that vaccine coverage does not provide dependable information on true population protection. Given this, a cost–benefit analysis of a serosurveillance sentinel network would be of great use.

The lowest levels of protection against measles virus were observed for the classes 0–1 and 1–2 in the 2 northern sites of our study. This is an interesting result given that half of the measles cases reported in the 2014 epidemic were very young (0–2 years old), and 90% of them were from the northern part of the country. Unfortunately, in the absence of information on the geographical origin of the patients from which the samples were taken, a direct relationship between seroprevalence and incidence is difficult to establish. Furthermore, the 4 sampling sites of the study happen by chance to be located in provinces of high incidence (Figure 3D), making such a relationship between seroprevalence and incidence even more difficult to unravel.

Two other details of the age–seroprevalence profiles observed in our study are the slow decrease of seroprevalence with age among teenagers and the fast increase of seroprevalence around the age of 20. As our seroepidemiological study is cross-sectional, it does not allow us to know whether the decrease of seroprevalence observed among teenagers is due to past increases in vaccine coverage or to some effect of waning immunity. Longitudinal data from a serosurveillance sentinel network would address questions such as these, thus, again, efficiently improving the development of vaccination and elimination strategies. As for the fast increase of seroprevelance around the age of 20 years, a likely explanation is the pre-wedding health checks that are routinely performed in Vietnam, which include updates of vaccination status.

Our logistic models show clear differences in seroprevalence by age and geographic location. The results confirm that our choice of sample size for each age class was adequately powered to detect effects. They also confirm that our sampling scheme was sufficiently balanced (despite a slight under-representation of females in Ho Chi Minh City) (Supplementary Table 1 and Supplementary Figure 2), as the LRT corrections for potential confounding effects did not lead to different conclusions from those not using corrections (results not shown).

In conclusion, our results suggest that a surveillance network of the levels of population protection based on the use of hospital residual serum samples could greatly improve the anticipation of epidemic risk and thus prevent epidemic by allowing for timely and adequately targeted (by age and location) interventions.

## Supplementary Data

Supplementary materials are available at *Open Forum Infectious Diseases* online. Consisting of data provided by the authors to benefit the reader, the posted materials are not copyedited and are the sole responsibility of the authors, so questions or comments should be addressed to the corresponding author.

ofz030_suppl_supplementary_figure_s1

ofz030_suppl_supplementary_figure_s2

ofz030_suppl_supplementary_figure_s3

ofz030_suppl_supplementary_tables
