# Supplementary figures and images for: Sero-Prevalence Surveillance to Predict Vaccine-Preventable Disease Outbreaks; A Lesson from the 2014 Measles Epidemic in Northern Vietnam
Source: Open Forum Infect Dis. 2019 Jan 24;6(3):ofz030. doi: 10.1093/ofid/ofz030 (PMC6405937; doi:10.1093/ofid/ofz030)

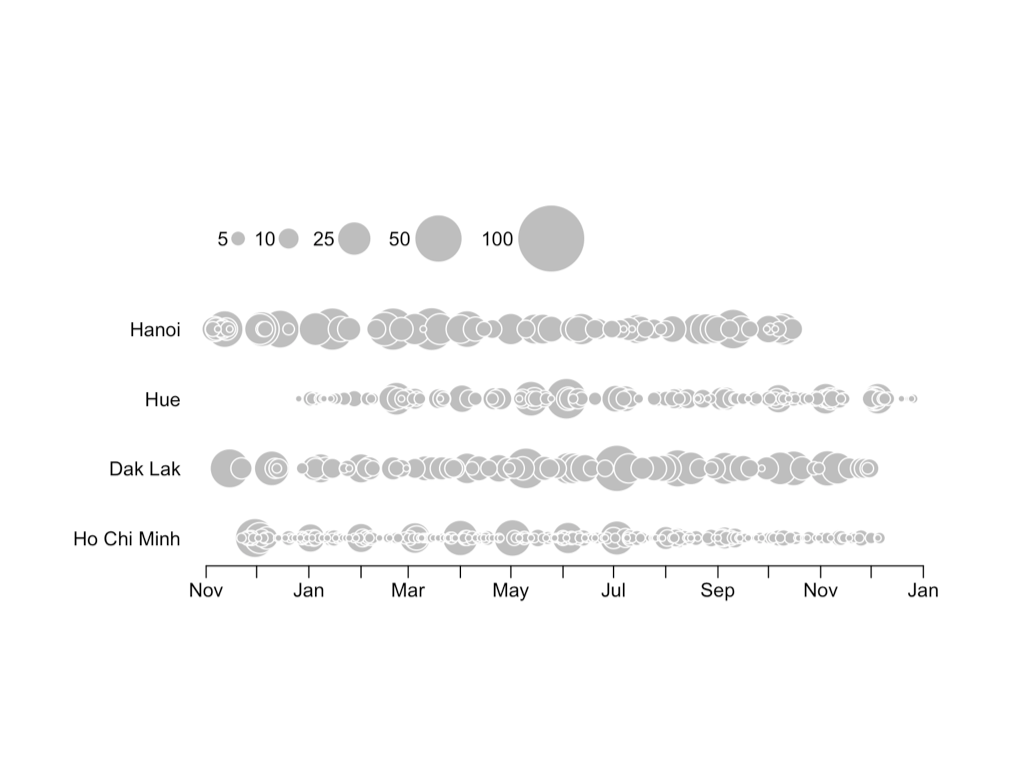

Supplement: ofz030_suppl_supplementary_figure_s1 [file ofz030_suppl_supplementary_figure_s1.png]

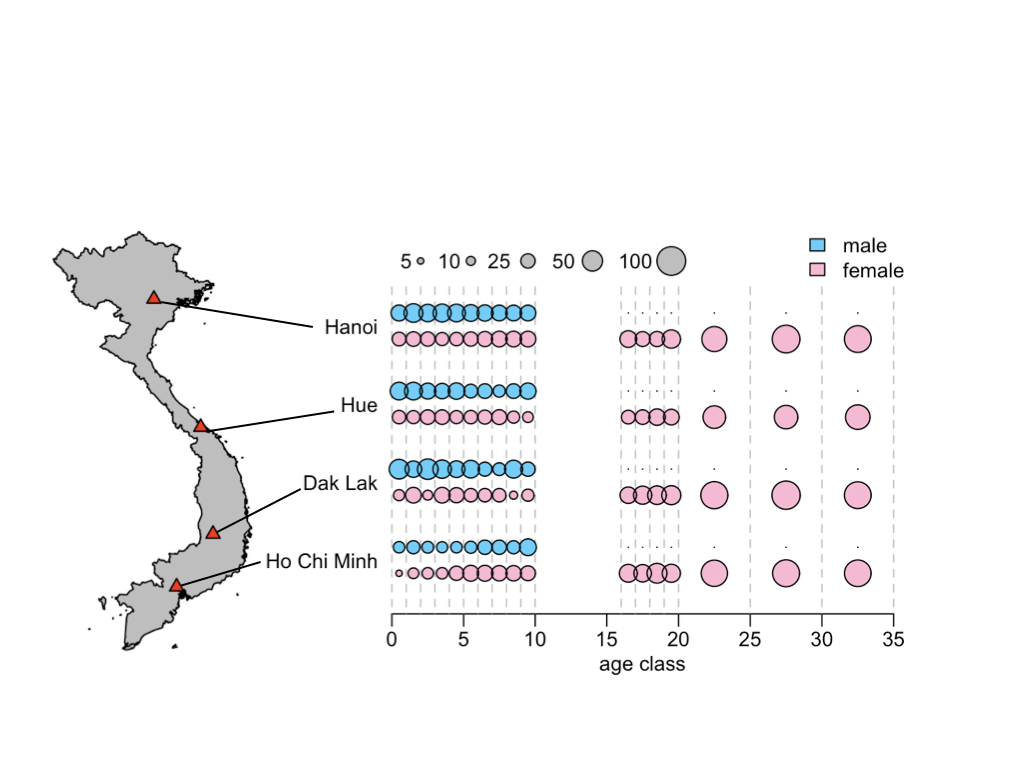

Supplement: ofz030_suppl_supplementary_figure_s2 [file ofz030_suppl_supplementary_figure_s2.png]

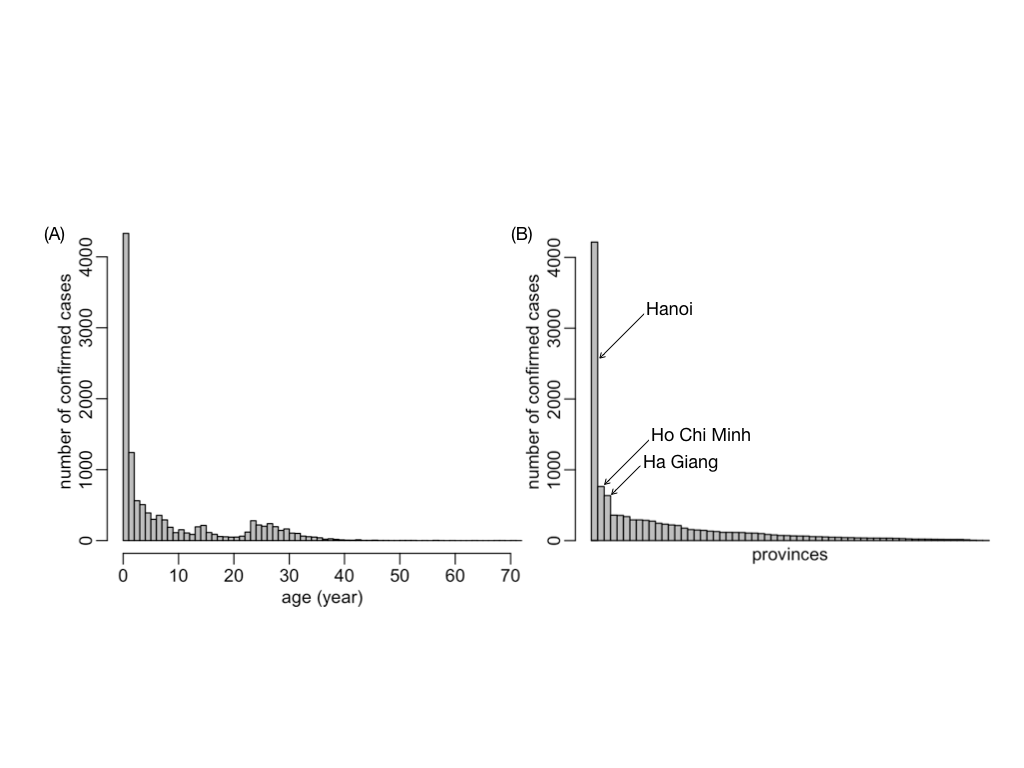

Supplement: ofz030_suppl_supplementary_figure_s3 [file ofz030_suppl_supplementary_figure_s3.png]
