# Supplementary material for: Sero-Prevalence Surveillance to Predict Vaccine-Preventable Disease Outbreaks; A Lesson from the 2014 Measles Epidemic in Northern Vietnam
Source: Open Forum Infect Dis. 2019 Jan 24;6(3):ofz030. doi: 10.1093/ofid/ofz030 (PMC6405937; doi:10.1093/ofid/ofz030)
Supplement: ofz030_suppl_supplementary_tables [file ofz030_suppl_supplementary_tables.docx]

**– Supplementary materials –**

Marc Choisy, Trinh Son Tung, Nguyen Thi Ngoc Diep, Nguyen Tran Hien, Mai Quynh Le, Pham Quang Thai, Tran Nhu Duong, Dang Duc Anh, Peter W. Horby, Maciej Boni, Juliet Bryant, Sonia O. Lewycka, Behzad Nadjm, H. Rogier van Doorn, Heiman F.L. Wertheim

Figures S1 and S2 show the samples split by time, location, age and gender. Figure S3 shows the age distribution of the measles cases during the 2014 epidemic, as well as its distribution per province. Table S1 shows similar information as Figure S2. Tables S2, S3 and S4 shows the MICS data for the questions IM3MD (measles vaccination information from the immunization card), HF13MD (measles vaccination information from the health facility) and IM16 (measles vaccination information from the mother) and how the different types of answers are interpreted in our study. Then, to compute the vaccine coverage we consider the following rules:

- any "vaccinated" value among IM3MD, IM16 or HF13MD corresponds to the presence of vaccination;
- among those who do not have a single "vaccinated" among IM3MD, IM16 or HF13MD, and "not vaccinated" for any of IM3MD, IM16 or HF13MD corresponds to the absence of vaccination;

the rest is considered as missing values (NA).

**Table S1:** number of processed samples per location, age class and gender (see also Figure S2).

|  | Hanoi | | Hue | | Dak Lak | | Ho Chi Minh | | Totals | | Total |
| --- | --- | --- | --- | --- | --- | --- | --- | --- | --- | --- | --- |
|  | ♂ | ♀ | ♂ | ♀ | ♂ | ♀ | ♂ | ♀ | ♂ | ♀ |  |
| 0-1 | 31 | 23 | 38 | 22 | 47 | 15 | 16 | 5 | 132 | 65 | 197 |
| 1-2 | 42 | 26 | 38 | 21 | 33 | 30 | 21 | 14 | 134 | 91 | 225 |
| 2-3 | 37 | 25 | 31 | 26 | 51 | 12 | 17 | 16 | 136 | 79 | 215 |
| 3-4 | 39 | 21 | 29 | 26 | 41 | 30 | 15 | 16 | 124 | 93 | 217 |
| 4-5 | 38 | 22 | 33 | 21 | 34 | 28 | 16 | 25 | 121 | 96 | 217 |
| 5-6 | 34 | 25 | 23 | 22 | 38 | 22 | 18 | 31 | 113 | 100 | 213 |
| 6-7 | 32 | 28 | 26 | 24 | 24 | 22 | 25 | 31 | 107 | 105 | 212 |
| 7-8 | 30 | 30 | 18 | 27 | 20 | 22 | 25 | 29 | 93 | 108 | 201 |
| 8-9 | 30 | 30 | 26 | 18 | 39 | 8 | 21 | 29 | 116 | 85 | 201 |
| 9-10 | 30 | 30 | 32 | 14 | 25 | 18 | 34 | 27 | 121 | 89 | 210 |
| 16-17 | – | 34 | – | 23 | – | 33 | – | 39 | – | 129 | 129 |
| 17-18 | – | 27 | – | 26 | – | 40 | – | 37 | – | 130 | 130 |
| 18-19 | – | 28 | – | 33 | – | 42 | – | 48 | – | 151 | 151 |
| 19-20 | – | 41 | – | 30 | – | 44 | – | 40 | – | 155 | 155 |
| 20-25 | – | 76 | – | 62 | – | 89 | – | 84 | – | 311 | 311 |
| 25-30 | – | 93 | – | 67 | – | 97 | – | 88 | – | 345 | 345 |
| 30-35 | – | 87 | – | 73 | – | 87 | – | 86 | – | 333 | 333 |
| < 11 | 343 | 260 | 294 | 221 | 352 | 207 | 208 | 223 | 1197 | 911 | 2108 |
| Total | 343 | 646 | 294 | 535 | 352 | 639 | 208 | 645 | 1197 | 2465 | 3662 |

**Table S2:** answers to the question IM3MD: day of measles vaccination from the immunization card. NA means missing value.

| answer | frequency | interpretation |
| --- | --- | --- |
| not given | 555 | not vaccinated |
| day of measles vaccination | 903 | vaccinated |
| marked on card | 2 | vaccinated |
| mother reported | 24 | vaccinated |
| inconsistent | 0 | NA |
| don’t know | 1 | NA |
| missing | 0 | NA |
| missing value | 1861 | NA |

**Table S3:** answers to the question HF13MD: day of measles vaccination from the health facility. NA means missing value.

| answer | frequency | interpretation |
| --- | --- | --- |
| not given | 570 | not vaccinated |
| vaccine shot | 1079 | vaccinated |
| marked on card | 8 | vaccinated |
| inconsistent | 0 | NA |
| don’t know | 0 | NA |
| missing | 2 | NA |
| missing value | 1687 | NA |

**Table S4:** answers to the question IM16: day of measles vaccination from the mother. NA means missing value.

| answer | frequency | interpretation |
| --- | --- | --- |
| vaccinated | 341 | vaccinated |
| not vaccinated | 169 | not vaccinated |
| don't know | 33 | NA |
| missing | 3 | NA |
| missing value | 2800 | NA |

**Figures legends**

**Figure S1:** number of samples collected in 2013 as a function of time and by sampling site (see figure 1A for the locations of the sites).

**Figure S2:** number of samples collected in 2013 by age class, gender and location (see figure 1A for the locations of the sites).

**Figure S3:** (A) age distribution of the measles cases during the 2014 measles epidemic. Age classes are 1-yr width. (B) Distribution of cases per province.
